# Cesarean or Vaginal Birth Does Not Impact the Longitudinal Development of the Gut Microbiome in a Cohort of Exclusively Preterm Infants

**DOI:** 10.3389/fmicb.2017.01008

**Published:** 2017-06-06

**Authors:** Christopher J. Stewart, Nicholas D. Embleton, Elizabeth Clements, Pamela N. Luna, Daniel P. Smith, Tatiana Y. Fofanova, Andrew Nelson, Gillian Taylor, Caroline H. Orr, Joseph F. Petrosino, Janet E. Berrington, Stephen P. Cummings

**Affiliations:** ^1^Alkek Center for Metagenomics and Microbiome Research, Department of Molecular Virology and Microbiology, Baylor College of Medicine, HoustonTX, United States; ^2^Newcastle Neonatal ServiceRoyal Victoria Infirmary, Newcastle upon Tyne, United Kingdom; ^3^School of Science and Engineering, Teesside UniversityMiddlesbrough, United Kingdom; ^4^Department of Statistics, Rice University, HoustonTX, United States; ^5^Faculty of Health and Life Sciences, Northumbria UniversityNewcastle upon Tyne, United Kingdom

**Keywords:** birth mode, cesarean, vaginal, gut microbiome, preterm infants, 16S rRNA sequencing

## Abstract

The short and long-term impact of birth mode on the developing gut microbiome in neonates has potential implications for the health of infants. In term infants, the microbiome immediately following birth across multiple body sites corresponds to birth mode, with increased *Bacteroides* in vaginally delivered infants. We aimed to determine the impact of birth mode of the preterm gut microbiome over the first 100 days of life and following neonatal intensive care unit (NICU) discharge. In total, 867 stool samples from 46 preterm infants (21 cesarean and 25 vaginal), median gestational age 27 weeks, were sequenced (V4 region 16S rRNA gene, Illumina MiSeq). Of these, 776 samples passed quality filtering and were included in the analysis. The overall longitudinal alpha-diversity and within infant beta-diversity was comparable between cesarean and vaginally delivered infants. Vaginally delivered infants kept significantly more OTUs from 2 months of life and following NICU discharge, but OTUs lost, gained, and regained were not different based on birth mode. Furthermore, the temporal progression of dominant genera was comparable between birth modes and no significant difference was found for any genera following adjustment for covariates. Lastly, preterm gut community types (PGCTs) showed some moderate differences in very early life, but progressed toward a comparable pattern by week 5. No PGCT was significantly associated with cesarean or vaginal birth. Unlike term infants, birth mode was not significantly associated with changes in microbial diversity, composition, specific taxa, or overall microbial development in preterm infants. This may result from the dominating effects of NICU exposures including the universal use of antibiotics immediately following birth and/or the lack of *Bacteroides* colonizing preterm infants.

## Introduction

Immediately following birth, a neonate encounters large numbers of viable microbes. Despite emerging evidence suggesting the potential for prenatal exposure to microorganisms during the fetal stages (Jiménez et al., 2008; Aagaard et al., 2014; Collado et al., 2016), the main colonisation event occurs at birth, where for term infants birth mode shapes what microbes are passed from the mother to the offspring (Aagaard et al., 2016). Within the first 24 h of life, the microbiome of multiple distinct sites across the neonate reflect the route of delivery, with vaginally delivered neonates harboring vaginally derived organisms (typically *Lactobacillus*) and cesarean delivered neonates harboring skin-like microbes (typically increased *Staphylococcus*) (Dominguez-Bello et al., 2010). In the subsequent days and weeks following birth, the microbes colonizing different body sites begin to show more distinction (Chu et al., 2017). In infants delivered at term, the impact of birth mode has been studied in several longitudinal studies, most showing infants delivered vaginally have increased *Bacteroides* throughout the 1st year of life (Azad et al., 2013; Jakobsson et al., 2014; Bäckhed et al., 2015; Bokulich et al., 2016; Yassour et al., 2016). Contrary to these reports, no difference in the overall microbial community or in specific taxa between vaginal and cesarean infants at 6 weeks of life has also been reported (Chu et al., 2017). Differences between cohorts and methods (e.g., sequencing depth) may account for these discrepancies.

In preterm infants, studies directly exploring associations between birth mode and the temporal microbiome are lacking, although evidence suggests other factors such as feeding practices, postnatal age, and diseases like sepsis or necrotizing enterocolitis (NEC) likely have a bigger association with the microbiome (Stewart et al., 2013a, 2015b, 2016; La Rosa et al., 2014; Cong et al., 2016; Hill et al., 2017). A recent meta-analysis in preterm infants found reduced Bacteroides and increased Firmicutes in cesarean infants (Pammi et al., 2017). Infants delivered by cesarean have increased risks of later life obesity (Yuan et al., 2016), allergy (Roduit et al., 2009), and asthma (Thavagnanam et al., 2008; Sevelsted et al., 2014). Notably, while the microbiome has been suggested to be involved in the pathobiology of these diseases, direct causality has not been demonstrated.

In the current study we combined our existing publically available datasets (Abdulkadir et al., 2016; Stewart et al., 2016) and our previously unpublished data to directly explore how the birth mode impacts the temporal development of preterm infants while on the NICU and following discharge.

## Materials and Methods

### Ethics Statement

Ethical approval was obtained from the County Durham and Tees Valley Research Ethics Committee. Parental written informed consent was given.

### Participants and Study Design

The study design, setting, participants, and methods of data collection have been reported previously (Stewart et al., 2012, 2013b; Abdulkadir et al., 2016). Briefly, all infants were cared for in a single NICU with standardized feeding, antibiotic and antifungal guidelines. Due to a change in NICU practice in 2013 infants born after this routinely received the probiotic Infloran^®^ (*Bifidobacterium bifidum*-ATCC15696 and *Lactobacillus acidophilus*-NCIMB701748) soon after initial introduction of feeds, where half an Infloran capsule was given twice daily (125 mg b.d. at 10 9 organisms per dose). Infants contributing a minimum of seven samples in their first 100 days were included in the study.

### 16S rRNA Gene Bacterial Profiling

Nucleic acid extraction was carried out on 100 mg of stool using the PowerLyzer^TM^ PowerSoil^®^ DNA Isolation Kit (MoBio, CA, United States) in accordance with the manufacturer’s instructions. The V4 region of the 16S rRNA gene was amplified by PCR using barcoded Illumina adapter-containing primers 515F and 806R (Caporaso et al., 2012) and sequenced on the MiSeq platform (Illumina; San Diego, CA, United States) by NU-OMICS using the 2 × 250 bp paired-end protocol yielding pair-end reads that overlap almost completely. Sequencing read pairs were demultiplexed based on the unique molecular barcodes, and reads were merged using USEARCH v7.0.1090 (Edgar, 2010). Merging allowed zero mismatches and a minimum overlap of 50 bases, and merged reads were trimmed at the first base with a Q ≤ 5. In addition, a quality filter was applied to the resulting merged reads and those containing above 0.05% expected errors were discarded. Sequences were stepwise clustered into OTUs at a similarity cutoff value of 97% using the UPARSE algorithm (Edgar, 2013). Chimeras were removed using USEARCH v7.0.1090. OTUs were determined by mapping the centroids to the SILVA database (Quast et al., 2013) containing only the 16S rDNA V4 region to determine taxonomies. A custom script constructed a rarefied OTU table (rarefaction was performed at only one sequence depth) from the output files generated in the previous two steps for downstream analyses. We utilized multiple quality control measures, including the use of non-template controls during microbial DNA extraction and 16S rRNA gene amplification. Resulting OTU tables were rarified to 4300 reads per sample.

### Bioinformatic and Statistical Analysis

Data analysis was conducted in R version 3.3 using ggplot2 (R Core Team, 2014). Alpha diversity analyses, specifically observed OTUs and Shannon diversity, are presented between infants. Weighted and unweighted UniFrac distances (Lozupone et al., 2011) were performed within infants based on consecutive samples. The number of OTUs kept (retained from one sample to the next), OTUs lost (present in the previous but not current sample), OTUs regained (any OTU detected in the current sample and previously detected within the infant, but not the preceding sample), and new OTUs gained (OTU detected in an infant for the first time) was performed within infants based on consecutive samples.

Inferred metabolic capacity of the bacterial community was determined by Tax4Fun (Asshauer et al., 2015). FishTaco was then performed at the pathway level using genomic content inference to determine which species attenuated and drove the significantly altered functions (Manor and Borenstein, 2017).

Preterm gut community types were determined based on a publically available script for linear mixed-effects modeling, medoid-based clustering, and Markov chain modeling (DiGiulio et al., 2015). Weighted UniFrac (Lozupone et al., 2011) was used to calculate the distance between all samples and this was denoised by extraction of the most significant PCoA eigenvectors before applying the PAM algorithm. Gap statistic was used to determine the appropriate number of clusters based on the section of the plot where the curve markedly flattens (i.e., the elbow phenomenon).

Cross-sectional analyses were performed at discrete time points (1, 3, 5, and 8 weeks of age) to overcome bias of repeated measures in longitudinal analyses. At a given time point, samples within ±10 week days included, where the sample nearest to the specific week were chosen, giving preference to samples collected prior to the time point. Significance of categorical variables were determined using the non-parametric Mann–Whitney test for two category comparisons or the Kruskal–Wallis test when comparing three or more categories (Kruskal and Wallis, 1952). All *P*-values were adjusted for multiple comparisons using the false discovery rate (FDR) algorithm (Benjamini and Hochberg, 1995). Linear regression models adjusted for age (day of life), sex, birth weight, gestational age, diagnosis of NEC and/or LOS, receipt of expressed breast milk, and antibiotics (< or >10 days of antibiotics while on the NICU).

## Results

### Study Population

In total, 63,592,993 sequencing reads from 867 samples (46 patients) mapped to the database, with 776 (760 NICU and 16 post discharge) samples remaining in the analysis following rarefication to 4300 reads. Of these 46 infants, 25 infants were delivered vaginally and 21 infants were delivery by cesarean section (**Table 1**). All infants received at least 48 h of antibiotics immediately following birth and the total number of days of antibiotic treatment while on the NICU was comparable between birth modes. Due to a change in unit practice, probiotics were administered in 4/21 of the cesarean infants born after 2013 and no vaginal infants received probiotics (*P* = 0.07). Use of probiotics was associated with increased relative abundance in only the genera contained within the probiotic (Infloran; *Bifidobacterium* and *Lactobacillus*), but not in other taxa (Supplementary Figure 1). Nonetheless, receipt of probiotics (along with other important covariates – see Materials and Methods) were also adjusted for in all significance testing of taxa.

**Table 1:** Characteristics of 46 preterm infants born by either vaginal or cesarean delivery.

	Vaginal	Caesarean	*P*-value
Infants	25	21	–
NICU samples^∗^	439	321	–
Post discharge samples^∗^	10	6	–
Birth weight in grams, median (IQR)	910 (750–1180)	950 (840–1150)	0.18
Gestational age in weeks, median (IQR)	26 (25–28)	27 (26–28)	0.18
Male sex	14 (56%)	16 (76%)	0.13
Received expressed breast milk	24 (96%)	20 (95%)	0.47
Received probiotics	0	4 (19%)	0.07
Number of days of antibiotics	8 (4–17)	6 (2–12)	0.35
NEC and/or LOS	7 (28%)	6 (29%)	1


*^∗^Samples remaining after QC and rarefaction.*

*NICU, neonatal intensive care unit; NEC, necrotising enterocolitis; LOS, late onset sepsis.*

### Longitudinal Alpha and Beta Diversity Was Comparable between Cesarean and Vaginally Delivered Infants

**FIGURE 1:**
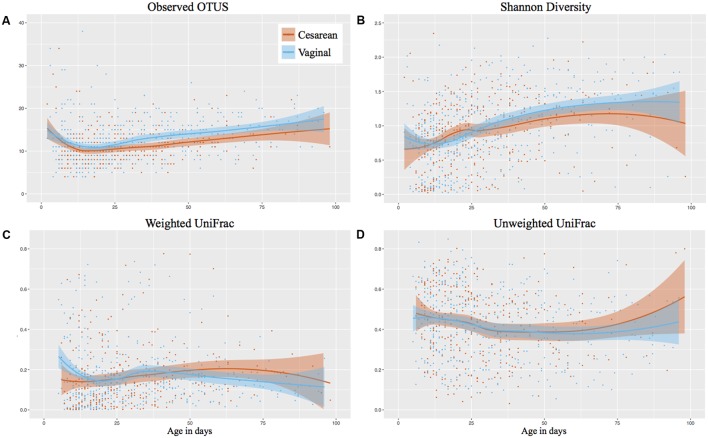
Longitudinal development of alpha and beta diversity by birth mode. Post discharge samples omitted from the analysis. Shaded bars represent the 95% confidence interval. **(A)** Observed OTUs (richness). **(B)** Shannon Diversity. **(C)** Weighted UniFrac based on consecutive samples. **(D)** Unweighted UniFrac based on consecutive samples.

The number of observed OTUs of samples decreased initially following birth, then increased from 11 OTUs on day 12 to 17 OTUs on day 100 of life. While vaginal infants had slightly more observed OTUs, there was no significant difference between cesarean and vaginally delivered infants (Mann–Whitney cross-sectional comparison at each week *P*-value was 0.43 or higher) (**Figure 1A**). The Shannon diversity increased from around 0.75 in the days following birth to 1.25 at NICU discharge, with comparable development between cesarean and vaginally delivered infants (Mann–Whitney cross-sectional comparison at each week *P*-value was 0.49 or higher) (**Figure 1B**). The observed OTUs and Shannon diversity continued to increase following discharge but no significant difference between cesarean and vaginally delivered infants occurred in post discharge samples (observed OTUs *P* = 0.212; Shannon *P* = 0.428) (Supplementary Figures 2A,B). Birth mode was also comparable between weighted and unweighted UniFrac distance between consecutive samples during NICU sampling (**Figures 1C,D**) and post discharge (weighted UniFrac *P* = 1; unweighted UniFrac *P* = 0.875) (Supplementary Figures 2C,D). Comparing the cross-sectional weighted UniFrac distance between birth modes at weeks 1, 3, 5, 8, and post discharge showed no significant difference at any time point (Supplementary Figure 3).

### Vaginally Delivered Infants Have Increased OTU Stability, But Comparable OTU Acquisition While on the NICU and Following Discharge

**FIGURE 2:**
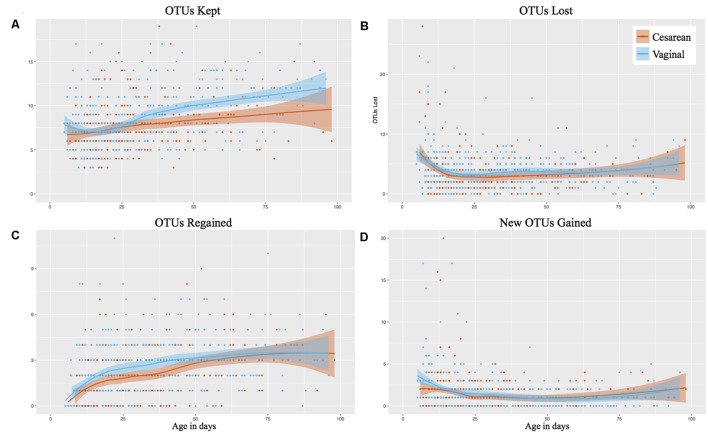
Longitudinal OTU tracker analysis of the gut microbiome in preterm infants by birth mode. Post discharge samples omitted from the analysis. Shaded bars represent the 95% confidence interval. **(A)** OTUs kept. **(B)** OTUs lost. **(C)** OTUs regained (previously detected in the infant). **(D)** New OTUs gained (not previously detected in the infant).

The individual OTUs were tracked through time in consecutive samples, showing that vaginally delivered infants kept significantly more OTUs from month 2 of life (*P* < 0.001) than those delivered by cesarean (**Figure 2A**). The number of OTUs kept in month 1 of life was comparable between birth modes (*P* = 0.947). The microbiome stabilized rapidly from birth to week 4 of life, where the number of ‘OTUs lost’ and ‘new OTUs gained’ declined, but the ‘OTUs regained’ (previously present but not in the preceding sample) increased (**Figure 2**). However, there was no difference in birth mode between OTUs lost, regained, or newly gained during the first 100 days while on the NICU. Following discharge, vaginally delivered infants continued to have significantly (*P* = 0.021) increased kept OTUs compared to cesarean, relative to the last NICU sample collected (Supplementary Figure 2E). As with the NICU samples, no significant difference between OTUs lost, regained, or newly gained was found for post discharge samples, relative to the last NICU sample (Supplementary Figures 2F–H).

### Bacterial Genera Were Comparable between Cesarean and Vaginally Delivered Infants

**FIGURE 3:**
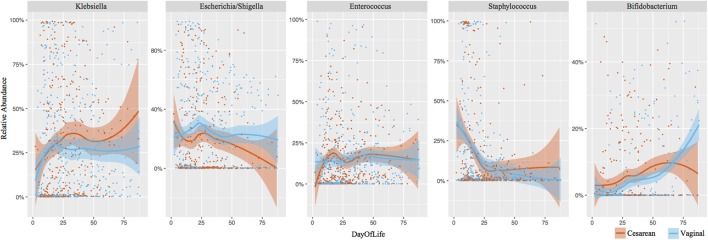
Longitudinal development of the dominant bacterial taxa by birth mode. Top five dominant taxa representing 84% of the overall relative abundance of NICU samples are shown. Each point represents an individual sample. Shaded bars represent the 95% confidence interval.

*Klebsiella* (28% overall relative abundance in NICU samples), *Escherichia* (22% overall relative abundance), *Enterococcus* (15%), *Staphylococcus* (14%), and *Bifidobacterium* (5%) dominated NICU samples, accounting for 84% of the total relative abundance in the first 100 days of life (**Figure 3**). *Klebsiella* and *Enterococcus* remained relatively consistent during the NICU period, *Staphylococcus* and *Escherichia* declined from birth, and *Bifidobacterium* gradually increased through the NICU period. To determine significant differences in the relative abundance of genera through the NICU, while accounting for repeated measures, the first sample from each infant in weeks 1, 3, 5, 8, and 10+ were included. This cross-sectional comparison showed no significant difference between cesarean and vaginal infants in any genera at any time point (Supplementary Table 1). *Bacteroides* was the 8th most abundant genera from all NICU samples (Supplementary Figure 4), but was 3rd most abundant in the post discharge samples (Supplementary Figure 5). Despite associations in term infants, the relative abundance of *Bacteroides* was comparable between cesarean and vaginal infants during NICU and following discharge in this preterm population. Comparing delivery mode in the post discharge period also showed the relative abundance of predominant genera was comparable (Supplementary Figure 5).

### Preterm Gut Community Type Development Is Comparable between Different Birth Modes

**FIGURE 4:**
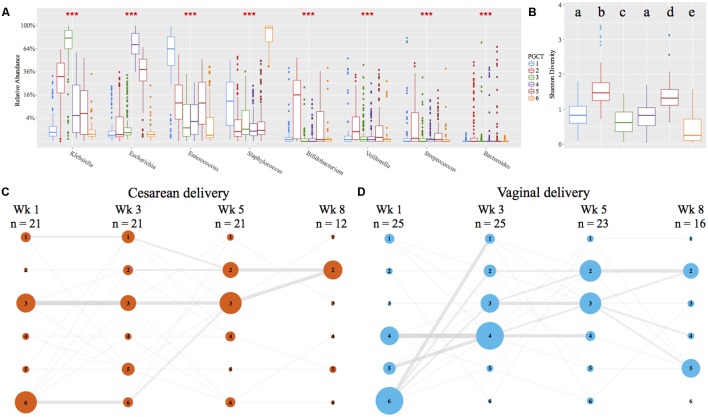
Preterm gut community type (PGCT) development in NICU samples. PGCT clustering based on weighted UniFrac. **(A)** Box plots showing the eight most significant genera between the six PGCTs. ^∗∗∗^ denotes a *P*-value of >0.001. **(B)** Box plots showing the Shannon Diversity of each PGCT. Lower case letters denote the significance, where a unique letter represents the Shannon diversity of that PGCT is significantly different from all other groups. Only ‘a’ exists more then once, demonstrating PGCT 1 and 4 are not significantly different to each other, but are significantly different to all other PGCTs. **(C,D)** PGCT transition model showing the progression of cesarean **(C)** and vaginal **(D)** infants through four distinct time points over the first 8 weeks of life. Nodes and edges are sized based on the total counts. See Supplementary Figure 6 for full trajectory plots of all samples.

Using Partitioning Around Medoids (PAM) clustering based on weighted UniFrac, the early preterm microbiome was defined by six distinct clusters, termed PGCTs (**Figure 4A**). With the exception of PGCTs 1 and 4, all PGCTs had significantly different Shannon diversity, with PGCTs 2 and 4 showing higher Shannon diversity and PGCT 6 (*Staphylococcus* dominant) showing the lowest Shannon diversity (**Figure 4B**). Birth mode showed moderate differences in the PGCTs detected over the initial weeks of life (**Figures 4C,D**). Most notable was the increase of PGCT 3 in cesarean delivery and PGCT 4 in vaginal delivery at week 1 of life. PGCT 4 continued to be increased in vaginal infants at week 3 of life, but by week 5 of life and thereafter the PGCTs detected in cesarean and vaginal infants were comparable. Despite these trends in PGCT development, the trajectory of gut microbiome development was highly variable within and between infants and no significant difference (*P* = 0.125) in PGCTs was found between cesarean and vaginal infants (Supplementary Figure 6).

### Inferred Bacterial Metabolic Potential Were Comparable between Cesarean and Vaginally Delivered Infants

Tax4Fun (Asshauer et al., 2015) was employed to infer the metabolic potential of the microbiome between birth modes. FishTaco (Manor and Borenstein, 2017) was performed to determine significance of inferred function at each time point (weeks 1, 3, 5, 8, and post discharge), finding no pathway was significantly altered between cesarean and vaginally delivered infants at any time point.

## Discussion

The role of birth mode on the initial acquisition and subsequent development of the infant microbiome remains an important research question. We investigated the longitudinal development of the microbiome during the first 100 days of life and following discharge. In a cohort of exclusively preterm infants (24–31 weeks gestation), sampled during NICU and post discharge, birth mode was not significantly associated with the alpha- or beta- diversity (both within and between patients). Furthermore, birth mode had no significant association with the relative abundance of any bacterial genera or PGCTs. A novel analysis temporally tracking OTUs within infants in consecutive samples showed vaginally delivered infants retain more OTUs from month 2 of life and post discharge, suggesting increased microbiome stability associated with vaginal delivery.

In a large meta-analysis of preterm gut microbiome, infants delivered by cesarean section had increased Firmicutes and reduced Bacteroides, but the overall microbiome profiles were comparable (Pammi et al., 2017). In a recent study of term and preterm infants, birth mode was associated with altered stool microbiome at weeks 1, 4, 8, and 24 for term infants only (Hill et al., 2017). While only four preterm infants delivered vaginally were included in the study by Hill et al. (2017), microbiome profiles of NICU and post discharge samples appeared comparable between the preterm infants born by cesarean or vaginal delivery. In a previous longitudinal study of 58 preterm infants, postconceptional age was associated with the gut microbiome, with delivery mode reported to have minimal influences (La Rosa et al., 2014). Furthermore, in a study of 29 preterm infants, gender and feeding were more associated with the gut microbiome development compared to other demographics, including birth mode (Cong et al., 2016). While no clear associations in preterm infants have been reported, birth mode has been associated with significantly altered gut microbiome in term infants in the 1st year of life (Azad et al., 2013; Jakobsson et al., 2014; Bäckhed et al., 2015; Bokulich et al., 2016; Yassour et al., 2016). The discrepancy between term and preterm infants may result from the greater use of antibiotics in preterm populations (Berrington et al., 2012), or other NICU practices that have a dominant effect on the microbiome. Specifically, the frequency with which preterm infants are considered to be at risk of early onset infection, and thereby determining the use of antibiotics for the first 48 h of life, may supersede perinatal maternal influences such as birth mode or reasons for preterm labor and/or expedited delivery. Alternatively, the difference may relate to the differential abundance of *Bacteroides* between term and preterm infants. *Bacteroides* is the primary genera significantly altered between cesarean and vaginal term infants over the 1st year of life (Azad et al., 2013; Jakobsson et al., 2014; Bäckhed et al., 2015; Bokulich et al., 2016; Yassour et al., 2016). However, *Bacteroides* was not dominant in the current study, nor previous studies of preterm populations during NICU (Taft et al., 2014; Stewart et al., 2015b; Ward et al., 2016; Warner et al., 2016; Hill et al., 2017). Following discharge, *Bacteroides* represented the third most abundant genera and was higher in vaginally delivered infants. However, low numbers of post discharge samples preclude robust analysis of significance.

Previous studies collecting samples immediately at birth have shown vaginally delivered term born neonates have increased *Lactobacillus* and cesarean delivered neonates have increased *Staphylococcus*, *Propionibacterium*, and *Streptococcus* across multiple body sites (Dominguez-Bello et al., 2010; Chu et al., 2017). The earliest sample collected in the current study was on day 2 of life, at which stage the infant had received antibiotics (most commonly penicillin and gentamicin). This may account for why no specific bacterial genera or PGCT was significantly associated with the birth mode. It is also noteworthy that samples collected within the initial hours of life generally reflect (viable and non-viable) microbes transmitted from the mother, and not true colonization *per se* (Aagaard et al., 2016). Thus, it is also possible that no difference was reported in the current cohort because the non-viable and non-colonizing organisms are no longer detected by day 2 of life. Furthermore, preterm infants have a greater exposure time to microbes in the NICU environment (e.g., surfaces, bedside equipment, and staff), which are also responsible for shaping the preterm microbiome (Brooks et al., 2014; Hartz et al., 2015), and might add to the discrepancies between the effects of birth mode on the developing microbiome between preterm and term infants.

In the current study a novel OTU tracker was applied to determine the OTUs kept, lost, gained, or regained through consecutive samples. This was the only analysis that found a significant association, showing that vaginal infants had an increased number of kept OTUs in consecutive NICU samples from month 2 of life, which remained in samples collected post discharge. This is potentially reflective of increased gut microbiome stability in vaginal infants, compared to cesarean (Bäckhed et al., 2015). No difference in kept OTUs was found in the 1st month of life, reflecting the dynamic nature of the microbiome in both cesarean and vaginal infants during this phase (Koenig et al., 2010; Stewart et al., 2016). Notably, the number of new OTUs gained while on the NICU was typically low (usually a single OTU per consecutive sample), but regained OTUs increased through NICU stay. This is likely a consequence of NICU practices, with limited environmental microbial exposure (e.g., use of sterile incubators, use of hand sanitizer, minimal skin-to-skin contact) restricting the introduction of new OTUs.

The current study has several limitations. First, a larger cohort comprised of preterm infants from multiple NICUs may reveal novel associations not seen in this single unit study. Second, collection of post discharge samples was challenging, with additional costs for sample collection and variable response rates to sampling requests, resulting in inclusion of post discharge samples from only 35% (16/46) of infants. Further study of post discharge samples is needed to determine the long-term associations of birth mode and specifically if *Bacteroides* establishes in higher relative abundance in vaginal infants (Azad et al., 2013; Jakobsson et al., 2014; Bäckhed et al., 2015; Bokulich et al., 2016; Yassour et al., 2016). Third, the universal use of antibiotics in this cohort prevents direct analysis on the role of antibiotics in superseding birth mode effects. Either much larger cohorts, or additional experimentation utilising animal models will be necessary to determine if antibiotics alone account for the discrepancies between preterm and term infants. Finally, the current analysis was performed only on 16S rRNA gene sequencing data, but differences in microbiome and host function may occur between vaginal and cesarean infants, requiring the use of proteomics and metabolomics to explore these elements further (Stewart et al., 2015a).

In summary, in a single NICU preterm population exposed to antibiotics postpartum, birth mode was not significantly associated with the diversity or composition of the microbiome. Vaginal infants tended to have greater stability following month 2 of life and post discharge. These findings highlight key differences between preterm and term infants. The long-term effects on host immune development from the transfer of microbes during delivery and the subsequent risk of obesity, autoimmunity, and allergy warrant further investigation.

## Author Contributions

CS, NE, GT, CO, JB, and SC were responsible for the study concept and design. CS and AN were responsible for extraction and sequencing of samples. CS, EC, PL, DS, TF, and JP were involved in the data processing and analysis. CS drafted the manuscript and all authors contributed to critical revisions and approved the final manuscript.

## Conflict of Interest Statement

The authors declare that the research was conducted in the absence of any commercial or financial relationships that could be construed as a potential conflict of interest.

## Abbreviations

LOS: late onset sepsis
NEC: necrotising enterocolitis
NICU: neonatal intensive care unit
OTU: operational taxonomic unit
PCoA: principal coordinates analysis
PGCT: preterm gut community type
