# Supplementary material for: Cesarean or Vaginal Birth Does Not Impact the Longitudinal Development of the Gut Microbiome in a Cohort of Exclusively Preterm Infants
Source: Front Microbiol. 2017 Jun 6;8:1008. doi: 10.3389/fmicb.2017.01008 (PMC5459931; doi:10.3389/fmicb.2017.01008)
Supplement: Supplementary file 1 [file Data_Sheet_1.DOCX]

Supplementary Material

**Cesarean or vaginal birth does not impact the longitudinal development of the gut microbiome in a cohort of exclusively preterm infants**

***Christopher J Stewart^1^, Nicholas D Embleton^2^, Elizabeth Clements^3^, Pamela N Luna^4^, Daniel P Smith^1^, Tatiana Y Fofanova^1^, Andrew Nelson^5^, Gillian Taylor^3^, Caroline Orr^3^, Joseph F Petrosino^1^, Janet E Berrington^2^, Stephen P Cummings^3^**

*** Correspondence:** Dr Christopher J Stewart: christopher.stewart@bcm.edu

**Sup Table 1. Multivariate linear regression with FDR corrected P values for top 5 most abundant genera following adjustment for covariates**

|  | Week 1 | Week 3 | Week 5 | Week 8 |
| --- | --- | --- | --- | --- |
|  | n = 46 | n = 46 | n = 44 | n = 28 |
| Klebsiella | 0.889 | 0.356 | 0.938 | 0.974 |
| Enterococcus | 0.635 | 0.227 | 0.938 | 0.974 |
| Escherichia | 0.889 | 0.410 | 0.938 | 0.974 |
| Bacteroides | 0.889 | 0.872 | 0.938 | NA |
| Bifidobacterium | 0.979 | 0.903 | 0.938 | 0.974 |
| Veillonella | 0.889 | 0.077 | NA | 0.974 |
| Actinomyces | 0.979 | NA | NA | 0.974 |
| Streptococcus | 0.979 | NA | NA | 0.974 |
| Staphylococcus | 0.990 | 0.978 | 0.938 | 0.974 |
| Acinetobacter | NA | 0.872 | NA | NA |
| Pseudomonas | NA | NA | 0.659 | 0.974 |
| Morganella | NA | NA | 0.838 | NA |
| Anaerococcus | NA | NA | NA | 0.244 |

Linear regression models adjusting for age (day of life), sex, birth weight, gestational age, diagnosis of necrotising enterocolitis and/or late onset sepsis, receipt of expressed breast milk, antibiotics (< or > 10 days of antibiotics while on the NICU). Only taxa included in >10% of samples at >0.1% mean abundance were included in the model.


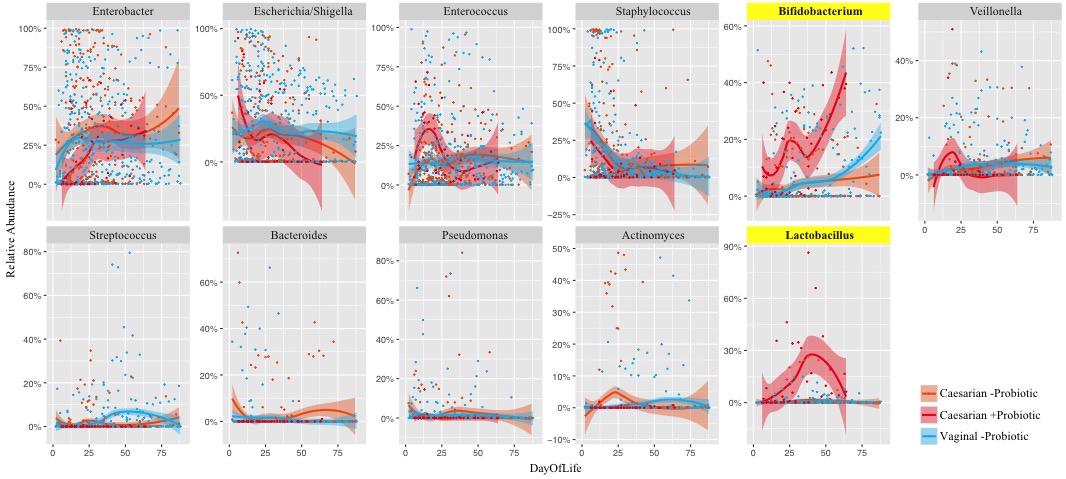


**Sup Fig 1. Longitudinal development of the top 11 most abundant genera by birth mode stratified by receipt of any probiotic**. The probiotic Infloran contained *Bifidobacterium bifidum*-ATCC15696 and *Lactobacillus acidophilus*-NCIMB701748 and the corresponding genera are highlighted in yellow. Receipt of probiotic only influenced *Bifidobacterium* and *Lactobacillus*. No vaginal infants received probiotics.

**Sup Fig 2. Box plots showing the alpha diversity, beta diversity, and OTU tracker results for post discharge samples by birth mode.** Low post discharge sample numbers (*n* = 6 cesarean and 10 vaginal) impede robust statistical comparisons.

**Sup Fig 3. Weighted UniFrac PCoA between cesarean and vaginally delivered infants.** Only a single sample from any given individual was included at each time point to avoid repeated measures. (**A**) Week 1. (**B**) Week 3. (**C**) Week 5. (**D**) Week 8. (**E**) Post discharge.


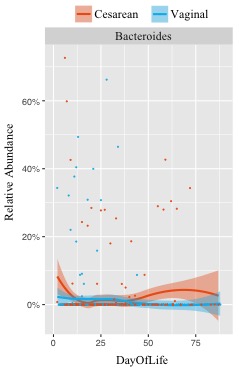


**Sup Fig 4. Temporal relative abundance of Bacteroides from samples obtained during NICU**

**Sup Fig 5. Box plots showing the top ten most abundant bacterial genera post discharge by birth mode**. Genera are ordered by overall relative abundance post discharge, where *Bifidobacterium* was most abundant. Low post discharge sample numbers (*n* = 6 cesarean and 10 vaginal) impede robust statistical comparisons.

**Sup Fig 6. Trajectory plots of the preterm gut community types (PGCTs) for each infant in the cohort.** All NICU samples included. PGCT clustering based on weighted UniFrac.
